# Reach and Weight Loss Among Comparison Group Participants Who Enrolled in the Active Intervention After a Diabetes Prevention Trial

**DOI:** 10.5888/pcd21.230358

**Published:** 2024-06-06

**Authors:** Tzeyu L. Michaud, Cleo Zagurski, Kathryn E. Wilson, Gwenndolyn C. Porter, George Johnson, Paul A. Estabrooks

**Affiliations:** 1 Department of Health Promotion, College of Public Health, University of Nebraska Medical Center, Omaha; 2 Center for Reducing Health Disparities, College of Public Health, University of Nebraska Medical Center, Omaha; 3 Department of Kinesiology and Health, College of Education & Human Development, Georgia State University, Atlanta; 4 Center for the Study of Stress, Trauma, and Resilience, College of Education and Human Development, Georgia State University, Atlanta; 5 Department of Health and Kinesiology, College of Health, University of Utah, Salt Lake City

## Abstract

We examined participation rates, engagement, and weight-loss outcomes of comparison group participants in a diabetes prevention trial who enrolled in a digitally delivered diabetes prevention program (ie, an active intervention) after the original trial ended. We evaluated these outcomes by using the Wilcoxon signed-rank test and 1-sample *z* test. We found a high participation rate (73%) among comparison group participants and comparable weight-loss outcomes at 12 months (6.8 lb) after initiating participation in the active intervention relative to intervention group participants during the original trial. Findings support providing evidence-based interventions for comparison or control group participants post-trial. Findings also support examining the cost-effectiveness of post-trial interventions, regardless of the limitations of acquiring post-trial data on weight in an uncontrolled setting.

**Summary**

**What is already known on this topic?**

Providing control or comparison participants with the opportunity to engage in an active intervention after completion of a randomized controlled trial is common practice in behavioral intervention research. Few data exist on participation rates, engagement, and effectiveness outcomes among control or comparison group participants post-trial.

**What is added by this report?**

Our study illustrates that participants in a comparison group who subsequently engaged in an active intervention experienced benefits similar to participants in the intervention condition of the original trial.

**What are the implications for public health practice?**

Integrating evidence-based interventions for control or comparison group participants should be considered in future behavioral programs and randomized controlled trials to enhance program reach and engagement.

## Objective

Approximately 97.6 million adults in the US have prediabetes (1). Results from a wide range of effectiveness trials demonstrate that the in-person or digitally delivered Diabetes Prevention Programs (DPPs) significantly reduce diabetes risk when compared with minimal or waitlist comparison groups (2–4). Many trials offer comparison group participants an opportunity to participate in an active intervention after the trial’s completion (5,6). The underlying assumption is that comparison group participants will engage with, and benefit from, the active intervention. However, few data exist on the participation rates, engagement, and effectiveness outcomes among comparison group participants post-trial. The objectives of this study were to 1) examine at 4-month and 12-month post-trial the reach and representativeness of comparison group participants in a diabetes prevention trial who, upon trial completion, enrolled and engaged in the active intervention; 2) document changes in weight at 4 months and 12 months post-trial; and 3) explore the magnitude of effect on weight loss by comparing the post-trial results of comparison group participants with the trial results of the intervention group participants.

## Methods

This pragmatic, observational study followed the Preventing Diabetes With Digital Health and Coaching for Translation and Scalability (PREDICTS) trial (4,7–10), which was conducted in Omaha, Nebraska, from December 2017 to March 2020. The randomized controlled trial (RCT) enrolled 599 overweight or obese adults with prediabetes (ie, hemoglobin A_1c_, 5.7%–6.4%), and randomly assigned participants to a digital DPP (n = 299) intervention group or small-group education (n = 300) comparison group. The 12-month DPP, delivered entirely digitally, is a translation of the original DPP lifestyle intervention and consisted of small-group support, personalized health coaching, digital tracking tools, and a weekly (initial 16 weeks) and monthly (subsequent 8 months) behavior-change curriculum (the Omaha Health Program) approved by the Centers for Disease Control and Prevention’s Diabetes Prevention Recognition Program (4,10). Comparison group participants were offered free enrollment in the digital DPP, similar to the intervention provided in the original trial, contingent upon the completion of the 12-month assessment session of the original trial. Of note, during the original trial, the intervention group lost significantly more body weight than the comparison group (12.2 lb vs 4.8 lb) (4).

During the post-trial period, the study team did not conduct any participant retention activities outside of strategies embedded in the digital DPP, and all data were collected pragmatically from participant surveys during the DPP enrollment process and from wireless home scales. The PREDICTS trial was approved by the University of Nebraska Medical Center and Western institutional review boards.

### Measures

Reach (11) was operationalized as the absolute number, proportion (eg, participation rate), and representativeness of comparison group participants enrolled in the digital DPP post-trial relative to the 1) total comparison group and 2) comparison group participants who completed the 12-month assessment of the original trial. Representativeness (11) was assessed by comparing demographic characteristics across groups of participants who, post-trial, enrolled in the digital DPP and completed 4-month or 12-month weigh-ins relative to the 1) total comparison group and 2) comparison group participants who completed the 12-month assessment of the original trial. Weight at trial completion was used as the post-trial baseline weight. We collected weights at 4 months and 12 months post-trial by using a wireless home scale, which automatically transmits the data to the server, provided as part of the digital DPP. 

### Statistical analysis

We compared sociodemographic and clinical variables between the comparison group participants who enrolled in the post-trial digital DPP and participants who did not enroll by using the nonparametric Kruskal–Wallis test for continuous variables and the Pearson χ^2^ test for categorical variables. We applied the Wilcoxon signed-rank (for continuous variables) and 1-sample *z* test of proportion (for categorical variables) to 1) examine group differences in demographic characteristics (ie, representativeness) and 2) compare the magnitude of weight loss at 4 months and 12 months post-trial of the comparison group participants who enrolled in the digital DPP to the weight loss at 4 months and 12 months in the original trial of the intervention group participants.

## Results

**Figure 1:**
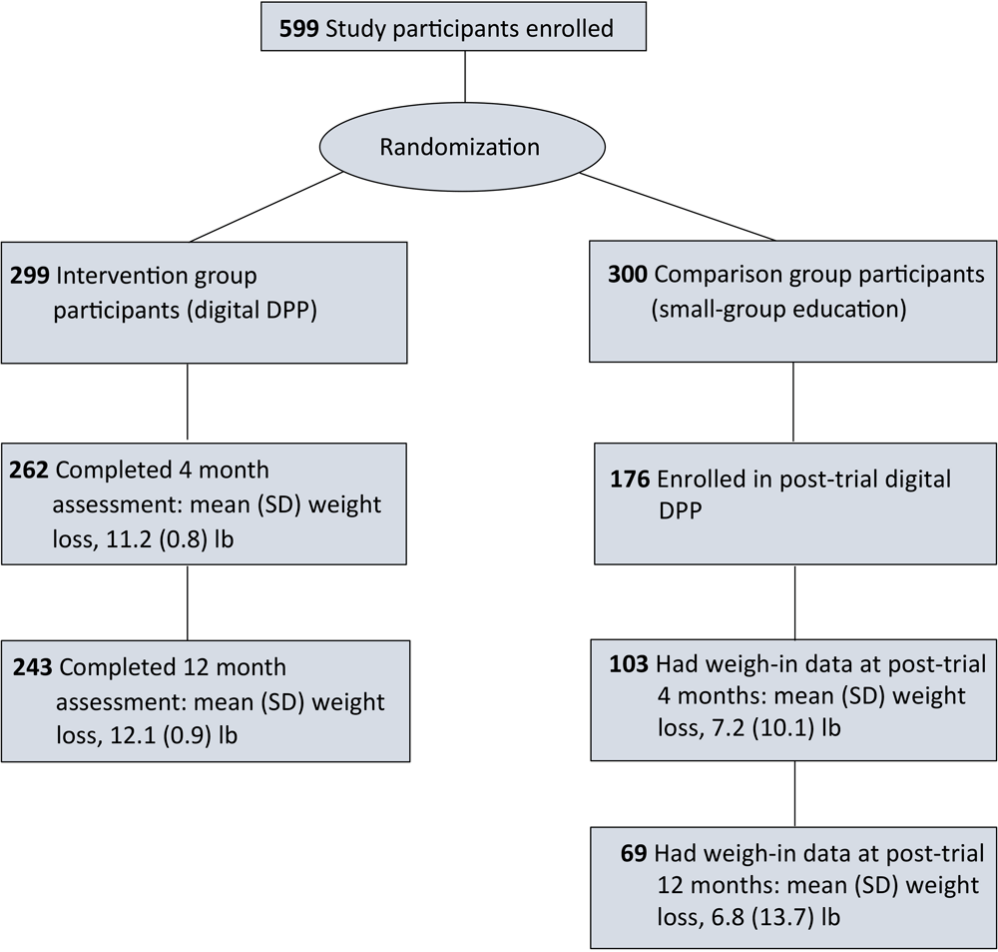
Flow diagram for adults with prediabetes participating in the Preventing Diabetes With Digital Health and Coaching for Translation and Scalability (PREDICTS) trial (4,7–10) and a post-trial Diabetes Prevention Program, Omaha, Nebraska, December 2017–March 2020.

Among 240 comparison group participants who completed the 12-month assessment in the original trial, 176 (73%) enrolled in the post-trial digital DPP (Figure 1). We found significant differences in age, sex, type of health insurance, low health literacy, and body mass index (BMI) between participants who enrolled in the post-trial digital DPP and those who did not enroll. Participants who enrolled were more likely than those who did not enroll to be women, have private health insurance, be younger, have lower health literacy scores, and have a higher BMI (Table 1). The characteristics of the comparison group participants who enrolled in the post-trial DPP were similar to the intervention group participants in the original trial (Appendix Table).

**Table 1:** Characteristics of Comparison Group Participants Who Enrolled in a Post-Trial Digital Diabetes Prevention Program, Omaha, Nebraska, 2020-2021

Characteristic	Enrolled (n = 176)	Did not enroll (n = 64)	Total (N = 240)	*P* value^a^
**Sociodemographic, no. (%)**				
Female	119 (68)	30 (47)	149 (62)	.01
White	154 (88)	59 (92)	213 (89)	.31
African American	17 (10)	4 (6)	21 (9)	.41
Hispanic or Latino	8 (5)	0	8 (3)	.18
Employment status				
Employed	119 (68)	37 (58)	156 (65)	.16
Retired	50 (28)	26 (41)	76 (32)	
Other	7 (4)	1 (2)	8 (3)	
Education attainment				
High school or less	22 (13)	12 (19)	34 (14)	.57
College (any)	101 (57)	36 (56)	137 (57)	
Advanced degree	52 (30)	16 (25)	68 (28)	
Annual household income, $				
<50,000	66 (38)	25 (39)	91 (38)	.71
50,000–100,000	30 (17)	7 (11)	37 (15)	
>100,000	58 (33)	23 (36)	81 (34)	
Missing	22 (13)	9 (14)	31 (13)	
Type of health insurance				
Medicare/Medicaid	42 (24)	27 (42)	69 (29)	.03
Private	123 (70)	35 (55)	158 (66)	
Other	7 (4)	2 (3)	9 (4)	
Low health literacy	14 (8)	10 (16)	24 (10)	<.001
Has hypertension	155 (88)	59 (92)	214 (89)	.36
**Clinical, mean (SD)**				
Age, y	55.7 (11.7)	59.5 (12.4)	56.7 (12.0)	.03
HbA_1c_, %	5.7 (0.3)	5.7 (0.3)	5.7 (0.3)	.61
Body weight, lb	224.4 (49.4)	217.8 (40.9)	222.8 (47.5)	.37
Body mass index, kg/m^2^	36.1 (7.0)	33.1 (4.7)	35.4 (6.6)	.003
HDL, mg/dL	50.0 (13.5)	49.3 (13.3)	49.8 (13.4)	.70
LDL, mg/dL	99.8 (33.8)	98.9 (32.4)	99.5 (33.3)	.86
Triglycerides, mg/dL	172.8 (108.8)	162.9 (76.0)	170.2 (101.2)	.51
PHQ-4 score^b^	1.9 (2.5)	2.0 (2.8)	1.9 (2.6)	.96
Perceived stress^c^	4.1 (3.0)	3.7 (3.2)	4.0 (3.0)	.36
Physical activity score^d^	27.8 (30.9)	37.0 (41.4)	29.8 (33.7)	.08
Dietary intake^e^	6.9 (2.7)	6.4 (2.1)	6.8 (2.6)	.18
Quality of well-being scores^f^	0.7 (0.1)	0.7 (0.1)	0.7 (0.1)	.32

Abbreviations: HbA_1c_, hemoglobin A_1c_; HDL, high-density lipoprotein; LDL, low-density lipoprotein; PHQ, patient health questionnaire.

a Group differences in sociodemographic and clinical characteristics were examined by using the nonparametric Kruskal–Wallis test for continuous variables and the Pearson χ^2^ test for categorical variables.

b Measured on a scale of 0 to 12, with higher values indicating more distress.

c Measured on a scale of 0 to 40, with higher values indicating higher perceived stress.

d Measured on a scale of 0 to 98, with higher values indicating higher physical activity engagement.

e Measured on a scale of 0 to 16, with lower values indicating a healthier diet.

f Measured on a scale of 0 to 25, with higher values indicating greater well-being.

### Representativeness and weight loss

The proportion of comparison group participants who were women at 4 months post-trial (74%) was greater than that of the total comparison group at trial baseline (61%) and comparison group participants who completed the 12-month trial (62%) (Table 2). Mean age was significantly higher among participants at 12 months post-trial compared with the total comparison group at trial baseline (59.2 y vs 55.6 y).

**Table 2:** Engagement of Comparison Group Participants Who Enrolled in the Post-Trial Digital Diabetes Prevention Program, by Assessment Time Points, Omaha, Nebraska, 2020-2021

Characteristic	Trial		Post-trial		
	Baseline (n = 300)	12 Months (n = 240)	Baseline (n = 176)^a^^,^ b	4 months (n = 103)^a^^,^ b ^,^ c	12 months (n = 69)^a^^,^ b ^,^ c
Female, no. (%)	184 (61)	149 (62)	119 (68)	76 (74)^d^^,^ e	49 (71)
White, no. (%)	269 (90)	213 (89)	154 (88)	93 (90)	62 (90)
African American, no. (%)	23 (8)	21 (9)	17 (10)	6 (6)	4 (6)
Hispanic or Latino, no. (%)	12 (4)	8 (3)	8 (5)	3 (3)	2 (3)
Age, mean (SD), y	55.6 (12.6)	56.7 (12.0)	55.7 (11.7)	57.1 (12.0)	59.2 (10.8)^d^
Body weight, mean (SD), lb^f^	229.1 (50.3)	222.8 (47.5)	224.4 (49.4)	212.5 (48.4)^d^^,^ g ^,^ e	207.0 (41.8) d ^,^ g ^,^ e
Weight change from post-trial baseline, mean (SD), lb^h^	—	—	—	−7.2 (10.1)	−6.8 (13.7)

a Values for group comparisons relative to the entire sample of comparison group participants at the trial baseline (n = 300) were examined by using 1-sample *z* tests of proportion for female, White, African American, and Hispanic or Latino, and Wilcoxon signed-rank test for age and body weight.

b Values for group comparisons relative to comparison group participants who completed trial 12 months (n = 240) were examined by using 1-sample *z* tests of proportion for female, White, African American, and Hispanic or Latino, and Wilcoxon signed-rank test for age and body weight.

c Values for group comparisons relative to comparison group participants at post-trial baseline (n = 176) were examined by using the 1-sample *z* tests of proportion for female, White, African American, and Hispanic or Latino, and Wilcoxon signed-rank test for age and body weight.

d Significant result for comparisons among comparison group participants at time points between post-trial 4 and 12 months and trial baseline.

e Significant result for comparisons among comparison group participants at time points between post-trial 4 and 12 months and trial 12 months.

f Average weight was calculated by using the available weight record at each time point.

g Significant result for comparisons among comparison group participants at time points between post-trial 4 and 12 months and post-trial baseline.

h Weight difference was calculated by using the available weight records at both time points. For example, the weight change at post-trial 4 months was calculated by including only participants who had weight-in data at both post-trial baseline and 4-month time points. Participants who had weigh-in data only at post-trial baseline were not included.

**Figure 2:**
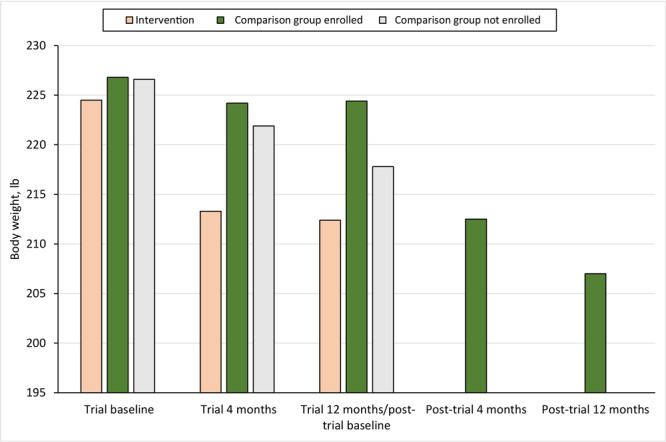
Weight-change outcomes among adults with prediabetes participating in the Preventing Diabetes With Digital Health and Coaching for Translation and Scalability (PREDICTS) trial (4,7–10) and a post-trial Diabetes Prevention Program, Omaha, Nebraska, December 2017–March 2020.

Among post-trial digital DPP participants, mean (SD) weight loss from post-trial baseline was 7.2 (10.1) lb at 4 months post-trial and 6.8 (13.7) lb at 12 months post-trial (Table 2). Weight loss was significantly different between the trial intervention group and the comparison group participants who enrolled in the post-trial digital DPP at 4 months post-trial (−11.2 lb vs −7.2 lb; *P* < .001) and 12-months post-trial (−12.1 lb vs −6.8 lb; *P* < .001) (Figure 2).

**Table:** 

Time point	Trial intervention group	Comparison group enrolled post-trial	Comparison group not enrolled post-trial
Trial baseline	224.5	226.8	226.6
Trial at 4 months	213.3	224.2	221.9
Trial at 12 months/post-trial baseline	212.4	224.4	217.8
Post-trial 4 months	—	212.5	—
Post-trial 12 months	—	207.0	—

## Discussion

In our study, to improve participant retention during the trial, we offered comparison group participants free enrollment in a digital DPP contingent upon the completion of the 12-month assessment session of the original trial. Although behavioral intervention research commonly uses wait-list control designs (for ethical reasons), offering an active intervention for control or comparison group participants post-trial as a retention strategy has rarely been reported. We found that comparison group participants who subsequently engaged in the active intervention had similar benefits to those in the intervention group of the original trial. However, the retention rates of participants in the digital DPP at 4 months (59%) and 12 months (39%) post-trial were inferior to the retention rates of intervention group participants (88% at 4 months and 81% at 12 months) in the original trial (4). This finding is unsurprising given the substantial resources invested in recruitment and retention efforts during the trial that were not invested in post-trial engagement for comparison group participants enrolled in the digital DPP (10).

By offering the active intervention after the trial ended, we found a temporal change in body weight among comparison group participants similar to that observed for participants during the original trial, especially among those who did not experience satisfying weight loss during the original trial. This benefit can be incorporated into future behavioral interventions and RCTs to enhance program reach (12). Additionally, we also noted that comparison group participants lost most of their weight during the initial few months of participation either during the original trial or post-trial periods. This observation may be attributable to interventions provided to these comparison group participants: either the 1-time 2-hour diabetes prevention class in a small-group format at the beginning of the trial or the intensive 16-week behavioral change curriculum focusing on weight loss during the post-trial (10).

In general, although weight-loss outcomes among control or comparison group participants are regularly reported during an RCT, data are seldom reported after cessation of the RCT. To the best of our knowledge, our study is the first to describe the potential benefits of expanding an active intervention to comparison group participants after RCT completion. Our results demonstrate comparable weight-loss outcomes among the comparison group post-trial relative to the intervention group in the original trial. Future studies should examine the costs associated with this strategy. At a rate of $742 per person for participation in the digital DPP (13) and an average weight loss at 12 months of 6.8 pounds, the average cost per pound of weight loss was $109 (or $241/kg), which is comparable to resource-intensive lifestyle modification programs ranging from $34 to $1,005, regardless of program duration (14,15). Additionally, the cost for this study was approximately $130,592 (176 × $742/person-year in 2020 US dollars), which may be conceptualized as part of the overall retention costs.

Limitations of this study relate to the acquisition of post-trial weight data, which were obtained from the self-weigh-ins via the wireless scale provided by the digital DPP in an uncontrolled setting (eg, the participant’s home) rather than obtained in a controlled setting and measured by research staff in the original trial. Moreover, weight changes at post-trial 4 and 12 months from post-trial baseline were calculated by using the available weight records at each time point (ie, per protocol analysis). Because the post-trial baseline weight among the 73 participants lacking post-trial 4-month weight data was higher than the post-trial baseline weight for the 103 participants who had the data (230 lb vs 220 lb), our results may have been susceptible to bias. However, we argue that even if participants did not weigh in at the post-trial 4-month, they still benefited from engaging in the active intervention. Despite these limitations, this study provides evidence and an assessment framework that is applicable to other behavioral interventions and to the examination of population-health cost-effectiveness.

## Appendix

**Table:** Characteristics of Comparison Group Participants Who Enrolled in the Post-Trial Diabetes Prevention Program (DPP) and Intervention Group Participants in the Original Trial^a^

Characteristic	Intervention group in the original trial (n = 299)	Comparison group enrolled in the post-trial DPP (n = 176)	*P* value^b^
**Sociodemographic, no. (%)**			
Female	184 (62)	119 (68)	.34
White	273 (91)	154 (88)	.14
African American	16 (5)	17 (10)	.08
Hispanic or Latino	7 (2)	8 (5)	.42
Employment status			
Employed	200 (67)	119 (68)	.53
Retired	80 (27)	50 (28)	
Other	19 (6)	7 (4)	
Education			
High school or less	37 (12)	22 (13)	.31
College (any)	195 (65)	101 (57)	
Advanced degree	66 (22)	52 (30)	
Annual household income, $			
<50,000	109 (37)	66 (38)	.83
50,000–100,000	46 (15)	30 (17)	
>100,000	99 (33)	58 (33)	
Missing	45 (15)	22 (13)	
Health insurance			
Medicare/Medicaid	97 (32)	42 (24)	.03
Private	195 (65)	123 (70)	
Other	6 (2)	7 (4)	
Has low health literacy	24 (8)	14 (8)	.14
Has hypertension	272 (91)	155 (88)	.32
**Clinical, mean (SD)**			
Age, y	55.3 (12.9)	55.7 (11.7)	.84
HbA_1c_, %	5.8 (0.3)	5.7 (0.3)	.56
Bodyweight, lb	224.5 (41.9)	224.4 (49.4)	.70
Body mass index, kg/m^2^	35.8 (6.1)	36.1 (7.0)	.24
HDL, mg/dL	48.5 (11.4)	50.0 (13.5)	.07
LDL, mg/dL	102.8 (31.6)	99.8 (33.8)	.98
Triglycerides, mg/dL	190.9 (104.0)	172.8 (108.8)	.72
PHQ-4 score^c^	2.0 (2.3)	1.9 (2.5)	.36
Perceived stress^d^	3.7 (2.7)	4.1 (3.0)	.17
Physical activity score^e^	17.3 (18.5)	27.8 (30.9)	.21
Dietary intake^f^	8.1 (2.5)	6.9 (2.7)	.30
Quality of well-being scores^g^	0.7 (0.1)	0.7 (0.1)	.23

Abbreviations: HbA_1c_, hemoglobin A_1c_; HDL, high-density lipoprotein; LDL, low-density lipoprotein; PHQ, patient health questionnaire.

^a^ The original trial is described elsewhere (4,7–10).

^b^ Determined by using the nonparametric Kruskal–Wallis test for continuous variables and the Pearson χ^2^ test for categorical variables.

^c^ Measured on a scale of 0 to 12, with higher values indicating more distress.

^d^ Measured on a scale of 0 to 40, with higher values indicating higher perceived stress.

^e^ Measured on a scale of 0 to 98, with higher values indicating higher physical activity engagement.

^f^ Measured on a scale of 0 to 16, with lower values indicating a healthier diet.

^g^ Measured on a scale of 0 to 25, with higher values indicating greater well-being.
